# Endotracheal intubation to reduce aspiration events in acutely comatose patients: a systematic review

**DOI:** 10.1186/s13049-020-00814-w

**Published:** 2020-12-10

**Authors:** Daniele Orso, Luigi Vetrugno, Nicola Federici, Natascia D’Andrea, Tiziana Bove

**Affiliations:** 1 Department of Medicine, University of Udine, P.le S. Maria della Misericordia 15, 33100 Udine, Italy; 2 Department of Anesthesia and Intensive Care Medicine, ASUFC University Hospital “Santa Maria della Misericordia” of Udine, Udine, Italy

**Keywords:** Glasgow coma scale, Intubation, Acute care, Aspiration, Outcome

## Abstract

**Background:**

It is customary to believe that a patient with a Glasgow Coma Scale (GCS) score less than or equal to 8 should be intubated to avoid aspiration. We conducted a systematic review to establish if patients with GCS ≤ 8 for trauma or non-traumatic emergencies and treated in the acute care setting (e.g., Emergency Department or Pre-hospital environment) should be intubated to avoid aspiration or aspiration pneumonia/pneumonitis, and consequently, reduce mortality.

**Methods:**

We searched six databases, Pubmed, Embase, Scopus, SpringerLink, Cochrane Library, and Ovid Emcare, from April 15th to October 14th, 2020, for studies involving low GCS score patients of whom the risk of aspiration and related complications was assessed.

**Results:**

Thirteen studies were included in the final analysis (7 on non-traumatic population, 4 on trauma population, 1 pediatric and 1 adult mixed case studies). For the non-traumatic cases, two prospective studies and one retrospective study found no difference in aspiration risk between intubated and non-intubated patients. Two retrospective studies reported a reduction in the risk of aspiration in the intubated patient group. For traumatic cases, the study that considered the risk of aspiration did not show any differences between the two groups. A study on adult mixed cases found no difference in the incidence of aspiration among intubated and non-intubated patients. A study on pediatric patients found increased mortality for intubated versus non-intubated non-traumatic patients with a low GCS score.

**Conclusion:**

Whether intubation results in a reduction in the incidence of aspiration events and whether these are more frequent in patients with low GCS scores are not yet established. The paucity of evidence on this topic makes clinical trials justifiable and necessary.

**Trial registration:**

Prospero registration number: CRD42020136987.

**Supplementary Information:**

The online version contains supplementary material available at 10.1186/s13049-020-00814-w.

## Introduction

It is customarily believed that a patient with an acute Glasgow Coma Scale (GCS) score of less than or equal to 8 should be intubated to avoid aspiration [1, 2]. Aspiration could lead to several complications, the main ones being aspiration pneumonia and pneumonitis. Aspiration pneumonia is derived from the invasion of the alveoli by gastric contents and subsequent bacterial growth. Pneumonitis is an inflammation of the alveoli, i.e., due to the gastric acid content. There are several risk factors for aspiration pneumonia: the loss of coordination of the swallowing muscles, e.g., due to dementia or neuromuscular diseases or the loss of airway protection reflexes, presumptively due to a reduced level of consciousness [3]. In fact, it is commonly believed that the reduction or abolition of the airways’ protective reflexes, i.e., the gag reflex, determines an essential favoring factor for aspiration.

Loss of consciousness can be caused by traumatic damage to the brain or by metabolic or toxic causes that cause a sedative effect. In a study involving 537 unconscious carbon monoxide poisoned patients, Sohn et al. found an incidence of aspiration pneumonia of about 19% [4].

Intubation could represent the safety of the airways, which are thus protected from the risk of aspiration. In a retrospective study by Fawcett et al. on 228 trauma patients, 89 (39%) had an aspiration event, 94% of cases occurred before intubation [5]. However, in a study conducted on non-traumatic unconscious patients, Nielsen et al. found that of 428 non-intubated patients, only 2 reported some complications, of which only one was an aspiration event [6].

An aspiration event could worsen the prognosis of patients. In the Fawcett et al. study, 16% of patients with an aspiration episode developed pneumonia (compared to 3.6% of patients with no aspiration event) [5]. However, the consequences of these complications do not seem to be univocal. Fawcett et al. did not detect an increase in mortality, ICU length of stay, or ventilation support [5]. Benjamin et al., in a retrospective study of 228 trauma patients, did not found any increased mortality for patients who experienced an aspiration episode [7].

Other studies observed an increased risk of hospitalization [8]. In addition, pre-hospital intubation can encounter complications related to the operator’s experience [9, 10] and the environment in which it takes place [11–13].

We aim to determine whether the orotracheal intubation of patients with a reduced level of consciousness (i.e., GCS ≤ 8) in the acute care setting (i.e., emergency department or pre-hospital setting) determines a lower risk of aspiration and related complications.

## Methods

We conducted a systemic review to establish if patients with GCS of ≤8 in the acute care setting require intubation to avoid aspiration. We divided the population into trauma and non-trauma patients. We used a systematic review method that allowed us to combine results of studies of different quantitative and qualitative methodology. In conducting the review, we followed the AMSTAR 2 publication standards for systematic reviews [14]. We searched six databases: Pubmed (1996–present), Embase (1974–present), Scopus (2004–present), SpringerLink (1950–present), Cochrane Library (1898–present), and Ovid Emcare (1995–present). We adopted the following keywords: “unconsciousness”, “Glasgow Coma Scale”, “aspiration”, “airway management”, “intubation”, and “Emergency” and applied them to the selected databases. The databases were reviewed from April 15th, 2019, to October 14th, 2020 (Supplementary Material).

### Data extraction

Two authors (NF and DO) recovered the full text of relevant articles. All related titles and abstracts were retrieved and searched for the full version. References from included studies and review articles were hand-searched to identify any additional relevant studies for analysis. Full-text papers were assessed initially for relevance and were subject to rapid appraisal using the Critical Appraisal Skills Programme (CASP) checklist [15]. Articles that do not meet the essential criteria for CASP (such as relevance to the review title) were excluded from further analysis.

#### Inclusion criteria

All observational or interventional studies (randomized controlled trials, brief reports, observational studies) that evaluated the risk of aspiration and the related complications (aspiration pneumonia and related mortality) in unconscious patients (evaluated by GCS) in the acute care setting (pre-hospital environment and emergency department) treated with orotracheal intubation (vs. no intubation) were included. No age limits were considered.

#### Exclusion criteria

We excluded non-English language studies, non-human studies or pre-clinical research, research protocols, policy statements, or guidelines. We excluded studies specifically on cardiac arrest patients at the scene in which cardiac arrest was the cause of intubation, since in these patients, the management of the airways does not play a prominent role as evidenced by the various international guidelines on the topic, and the risk of aspiration is subordinate to the restoration of cardiopulmonary function.

We extracted and reported the following data for each of the studies included in the review: publication year, type of population, enrollment, method, sampling, setting, sample size, inclusion criteria, qualification of the operator, and the measured outcome.

The protocol of the current review was registered in Prospero: CRD42020136987.

### Quality appraisal

Assessment of the considered articles’ quality was undertaken using the CASP (Critical Appraisal Skills Programme) checklist. Two independent reviewers (DO and NF) read all papers and scored them according to the CASP checklist [15]. Any disagreement was discussed between the two reviewers. If no agreement was reached after the discussion, a third author (LV) was involved. The studies which passed the quality selection by reviewers were considered in the systematic review. An agreement between two out of three reviewers was considered sufficient to include the disputed study.

### Presentation of results

Due the included studies’ heterogeneity, we considered a quantitative synthesis (meta-analysis) not feasible. We summarized the evidence from the literature by presenting the results of the individual studies included.

## Results

**Fig. 1:**
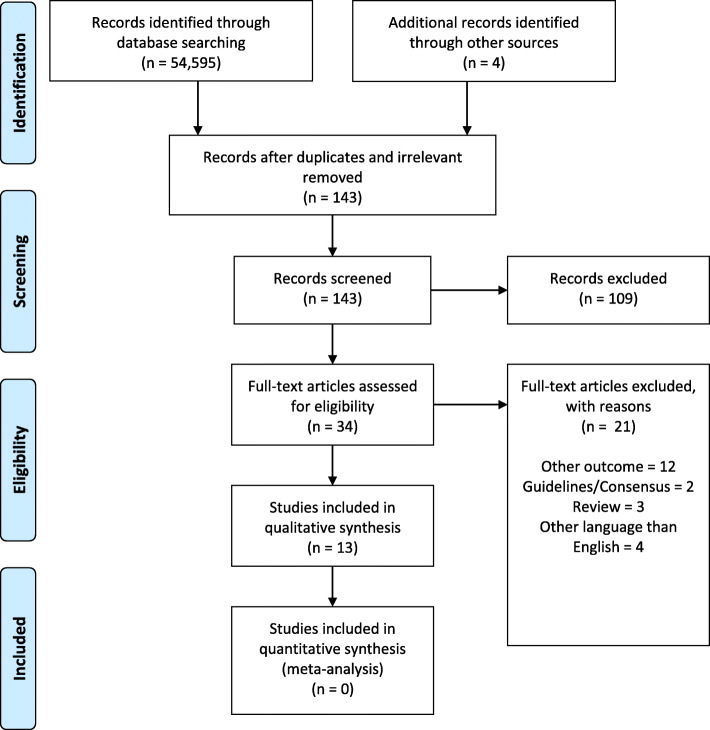
Flow diagram of literature search and study selection

The selection strategy flowchart is shown in Fig. 1. Our search found 54,599 records; we considered 143 abstracts after removing irrelevant and duplicated titles. Finally, 34 publications underwent a full paper review, and 13 studies were included in the final analysis. The characteristics of the considered studies are shown in Table 1. Publication dates range from 1991 to 2019. Seven studies involved non-traumatic unconscious patients (poisoned or intoxicated patients) [6, 8, 16–20], 4 studies enlisted traumatic brain injury (TBI) patients [11, 21–23]. A study enrolled adult patients who required intubation for traumatic and non-traumatic cases [24]; a study enrolled pediatric mixed traumatic and non-traumatic cases [12].

**Table 1:** List of the studies included in the systematic review and their characteristics

Study	Population	Method	Enrollment	Sampling	Setting	Sample size	Inclusion criteria	Operators	Outcome	Results
***Non-traumatic patients***										
*Donald* [16] *2009*	Poisoned patients	Observational	Prospective	Non consecutive	ED	26	GCS ≤ 8	ED physicians	- Aspiration - Hospital LOS - Mortality	- Aspiration: none - Hospital LOS: non-intubated group vs. intubated group (26 h vs. 5.4 days) - Mortality: non-intubated group vs. intubated group (0 vs 1)
*Duncan* [8] *2009*	Poisoned patients	Observational	Prospective	Consecutive	ED	73	GCS ≤ 15	ED physicians	- Aspiration - Admission to ICU	- Aspiration: none - ICU admission: 1 in GCS ≤ 8 group - Hospital LOS: GCS ≤ 8 vs GCS > 8 (26 h vs 14 h)
*Eizadi-Mood* [17] *2009*	Poisoned patients	Observational	Retrospective	n.a.	ED	155	GCS ≤ 12	ED physician	- Aspiration pneumonitis	- Aspiration pneumonitis: intubation (OR = 0.07; 95%CI = 0.01–0.49) [GCS (OR = 0.43; 95%CI = 0.30–0.62)]
*Montassier* [18] *2012*	Poisoned patients	Observational	Prospective	Consecutive?	PH and ED	72	GCS ≤ 8	PH and ED physicians	- Aspiration pneumonia	- Aspiration pneumonia: 9/13 (69.2%) in delayed ED intubation group vs. 6/34 (17.7%) ED intubation group (*p* = 0.002). [GCS > 8 group vs GCS ≤ 8 group (6 [24%] vs 15 [31.9%]; *p* = 0.48)]
*Nielsen* [6] *2012*	Non-traumatic unconscious patients	Observational	Retrospective	n.a.	PH	557	GCS < 9	PH physicians	- Intubation during hospitalization	- 64/557 (11%) patients remained unconscious to ED; 12 (2%) of these were intubated in the ED
*Dietze* [19] *2014*	Gamma-hydroxybutyric acid overdose patients	Observational	Retrospective	n.a.	ED	335	GCS ≤ 8	ED physicians	- ED LOS - Admission status	- ED LOS: intubated vs. non-intubated group + 41% (Exp B = 1.41, 95%CI = 1.19 to 1.65) - Probability of hospital admission: intubated vs. non-intubated group (OR = 0.10, 95%CI = 0.02 to 0.65)
*van Helmond* [20] *2019*	Gamma-hydroxybutyric acid overdose patients	Observational	Retrospective	n.a.	ED	158	GCS < 9	PH and ED physicians?	- Aspiration	- Major aspiration events were reported in 5 patients (2.4%; 95%CI: 0.8–5.5) [2 required intubation (16.7%; 95%CI: 2.1–48.4)]
***Trauma patients***										
*Davis* [21] *2005*	TBI	Observational	Prospective	Consecutive?	PH	412	GCS ≤ 8	EMS providers	- Pre-RSI hypoxia - Aspiration - Mortality	- Hypoxia: before RSI vs. after RSI (23.9% vs. 5.9%, OR 4.97, 95%CI = 3.07–8.07, *p* = 0.001) - Aspiration: GCS ≤8 (χ^2^ *p* = 0.14; AUC = 0.55) - Mortality: GCS ≤8 (χ^2^ *p* < 0.001 AUC = 0.58)
*Irvin*^*11*^ *2010*	TBI	Observational	Retrospective	n.a.	PH	10,948	GCS = 3	EMS providers?	- Mortality	- Mortality rate: hospital intubated vs. prehospital intubated group (35% vs. 62%; p < 0.0001)
*Evans* [22] *2011*	TBI	Observational	Retrospective	n.a.	PH	197	TBI needed intubation	EMS providers?	- Pneumonia	- GCS score in pneumonia group vs. non-pneumonia group (7.9 ± 0.9 vs 9.9 ± 0.4 p = 0.04) - Use of BVM in pneumonia group vs. non-pneumonia group (18 [56.3%] vs. 56 [34.0%]; p = 0.02)
*Emami* [23] *2019*	TBI	Observational	Retrospective	n.a.	PH	21,242	GCS < 9	PH physicians	- Mortality	- Mortality: intubated vs. non-intubated group (42.2% vs. 30.0%, respectively). In patients < 15 years old (42.2% vs. 33.4%; *p* = 0.03)
***Adult Mixed cases***										
*Steuerwald* [24] *2018*	Mixed adult patients	Observational	Retrospective	n.a.	PH	161	Adults patients who required airways management	EMS providers	- Aspiration	- Aspiration: 59 cases (8%) in supraglottic device group vs. 91 cases (12%) in endotracheal tube group (*p* value = .359; relative risk = .841; 95%CI .329–2.152)
***Pediatric Mixed cases***										
*Tweed* [12] *2018*	Pediatric unconsciuous patients	Observational	Retrospective	n.a.	PH	104	Unconscious pediatric patients	EMS providers	- Oxygenation - Mortality	- Inadequate oxygenation: 4% in intubated group; 9% in BVM patients; 32% in only oxygen group. - Mortality: Trauma patients: intubated vs. non-intubated group (RR 0.61 [95% CI, 0.35–1.06]; *P* = .082). Non-traumatic patients: intubated vs. non-intubated group (RR 2.98 [95% CI, 1.18–7.56]; *P* = .021)

*N.a.* Not available, *ED* Emergency Department, *n.s.* Not specified, *TBI* Traumatic brain injury, *PH* Pre-hospital, *RSI* Rapid sequence intubation, *ICU* Intensive care unit, *LOS* Length of stay, *EMS* Emergency medical system, *GCS* Glasgow Coma Scale, *χ2* Chi-square, *p p*-value, *OR* Odds ratio, *CI* Confidence interval, *AUC* Area under the curve, *h* Hours, *BVM* Bag valve mask, *Exp B* Coefficient for the linear regression

All studies were observational: 4 enrolled patients prospectively [8, 16, 18, 21], while the remaining 9 were retrospective [6, 11, 12, 17, 19, 20, 22–24]. Only one study, among those prospective, specified the consecutive enrollment of patients [8]. Five studies were conducted in an Emergency Department (ED) [8, 16, 17, 19, 20], 7 related to the pre-hospital environment [6, 11, 12, 21–24], and one study was conducted both in the pre-hospital setting and in the ED. [18] The sample size ranged from 26 to 21,242 patients. Most of the studies specified inclusion criteria, i.e., a GCS less than or equal to 8. Some studies did not specify this criterion, or enrolled any patient with a GCS less than 15 (then classifying them into subgroups). The role of the operator who performed the intubation was, in 7 studies, a physician (in most cases an ED physician) [5, 8–12, 17]; in 4 studies, it was an EMS provider (in most cases a paramedic) [11, 21, 22, 24].

### Quality appraisal

We evaluated the included studies as high, moderate, and low-reliability (Table 2). The average reliability was moderate or low. Many studies were retrospective, so the risk of bias not precisely assessable [6, 11, 12, 17–24]. We did not identify any randomized clinical trials. Only 4 studies included a control group of non-intubated patients [6, 8, 16, 24].

**Table 2:** Assessment of quality and reliability of the individual studies

Study	Section A: Are the results of the study valid?						Section B: What are the results?			Section C: Will the results help locally?
	Did the study address a focused issue?	Was the cohort recruited acceptably?	Was the exposure accurately measured to minimize bias?	Was the outcome accurately measured to minimize bias?	Have the authors identified all-important confounding factors?	Was the follow up of subjects complete enough?	Have ethical issues been taken into consideration?	Was the data analysis sufficiently rigorous?	Is there a clear statement of findings?	How valuable is the research?
***Non-traumatic patients***										
Donald [16] 2009	*Yes*	*Yes*	*Uncertain*	*Yes*	*Uncertain*	*Yes*	*Yes*	*Yes*	*Yes*	*High*
Duncan [8] 2009	*Yes*	*Yes*	*Uncertain*	*Yes*	*Uncertain*	*Yes*	*Yes*	*Yes*	*Yes*	*High*
Eizadi-Mood [17] 2009	*Yes*	*Uncertain*	*Uncertain*	*Uncertain*	*Uncertain*	*Yes*	*Yes*	*Yes*	*Yes*	*Moderate*
Montassier [18] 2012	*Yes*	*Yes*	*Uncertain*	*No*	*Uncertain*	*Uncertain*	*Uncertain*	*Uncertain*	*Yes*	*Low*
Nielsen6 2012	*Yes*	*Yes*	*Uncertain*	*No*	*No*	*Yes*	*Yes*	*Yes*	*Yes*	*Low*
Dietze [19] 2014	*Yes*	*Uncertain*	*Uncertain*	*Yes*	*Uncertain*	*Yes*	*Yes*	*Yes*	*Yes*	*Low*
Van Helmond [20] 2019	*Yes*	*Yes*	*Uncertain*	*Yes*	*Uncertain*	*Yes*	*Yes*	*Yes*	*Yes*	*Moderate*
***Trauma patients***										
Davis [21] 2005	*Yes*	*Yes*	*Uncertain*	*Yes*	*No*	*Yes*	*Yes*	*Yes*	*Uncertain*	*Moderate*
Irvin [11] 2010	*Yes*	*Uncertain*	*Uncertain*	*Uncertain*	*Uncertain*	*Yes*	*Yes*	*Yes*	*Yes*	*Moderate*
Evans [22] 2011	*Yes*	*Uncertain*	*Uncertain*	*Yes*	*Uncertain*	*Yes*	*Yes*	*Yes*	*Yes*	*Moderate*
Emami [23] 2019	*Yes*	*Uncertain*	*Uncertain*	*Yes*	*Uncertain*	*Yes*	*Yes*	*Yes*	*Yes*	*Moderate*
***Adult Mixed cases***										
Steuerwald [24] 2018	*Yes*	*Uncertain*	*Uncertain*	*Yes*	*Uncertain*	*Yes*	*Yes*	*Yes*	*Yes*	*Moderate*
***Pediatric Mixed cases***										
Tweed [12] 2018	*Uncertain*	*Uncertain*	*No*	*Uncertain*	*Uncertain*	*Yes*	*Yes*	*Uncertain*	*Yes*	*Low*

In most studies, an “unconscious” patient has defined if the GCS score was less than or equal to 8; however, some exceptions used different GCS values as cut-off [8, 17].

Among the studies, there was no agreement for the outcome considered. Some studies evaluated the cases of aspiration complication (pneumonia or pneumonitis) [8, 16, 17, 20, 21, 24], other studies considered a clinical outcome [8, 11, 12, 16, 19, 21, 23], like mortality, ICU length of stay, or hospital length of stay, related to an aspiration event.

### Summary of results

A summary of the results has been provided in Table 1.

#### Non-traumatic patients

Regarding the studies involving non-traumatic patients, two prospective observational studies did not find any difference in aspiration pneumonia between unconscious intubated and non-intubated patients [8, 16]. In Eizadi-Mood et al. retrospective study, patients who received intubation accounted for 30% of aspiration pneumonia cases, while the remaining 70% were in non-intubated patients (relative risk = 3.35; 95% CI = 1.33–8.48; *p* = 0.008). Intubation was found to be protective for aspiration pneumonia (odds ratio = 0.07; 95% CI = 0.02–0.49) [17]. In the study by Montassier et al., 9 of the 13 (69%) patients who were intubated late in the ED developed aspiration pneumonia, compared to 6 of the 34 (18%) patients who underwent immediate intubation upon arrival in the ED (*p* = 0.002) [18]. In this study, a GCS grade < 8 was not a variable associated with an increase in aspiration pneumonia incidence (*p* = 0.48).

The studies that considered mortality for non-traumatic unconscious patients did not find an increase in this outcome in the group of unconscious patients not subjected to intubation [8, 16].

#### Traumatic patients

Regarding the studies conducted on patients with TBI, only the study by Davis et al. considered the risk of aspiration specifically, noting no difference between intubated and non-intubated patients [21].

Almost all studies in traumatic patients considered mortality as an outcome. Davis et al. found a statistically significant association between low GCS scores and mortality, regardless of intubation procedure (OR = 0.3; 95%CI = − 0.1 – 0.8) [21]. Emami et al. found an increase in mortality in unconscious intubated patients in the pre-hospital setting compared to non-intubated patients (42.2% vs. 30.0%) [23]. Irvin et al. found an increase in mortality among intubated patients in the pre-hospital compared to those intubated in the ED only (62% vs. 35%; *p* < 0.0001) [11].

Finally, Evans et al. found a statistically significant association between low GCS scores, bag-mask ventilation, and ventilator- associated pneumonia incidence (7.9 ± 0.9 vs 9.9 ± 0.4; *p* = 0.04 and 56.3% vs 34.0%; *p* = 0.02 respectively) [22].

#### Adult mixed cases

A study conducted in a pre-hospital setting compared the incidence of aspiration events (diagnosed by radiological imaging) among a group of mixed adult patients (both trauma and non-trauma patients) treated with a supraglottic device or with orotracheal intubation. The Authors found no statistically significant difference [24]. The heterogeneity of the cases and the fact that the rescue personnel had sedated some patients should be noted.

#### Pediatric mixed cases

The study by Tweed et al. considered traumatic (55%) and non-traumatic (45%) pediatric patients managed in the pre-hospital environment. The causes of a non-traumatic state of unconsciousness were seizure (48%), respiratory (32%), drowning (15%), cardiac/respiratory arrest (12.5%), altered loss of consciousness (8%).

The authors did not detect the incidence of aspiration phenomena. However, they concluded that, while for traumatic patients, there is no correlation between orotracheal intubation and mortality; for non-traumatic patients, stratified by GCS level, there is an increase in intubated patients’ mortality compared to non-intubated patients [12].

## Discussion

The studies we have considered present conflicting results. Regarding the primary outcome considered, i.e., the risk of aspiration, this seems different depending on the type of patient considered. For studies on non-traumatic patients, the prospective studies did not show a significant difference in aspiration risk between intubated and non-intubated patients. On the other hand, in retrospective studies, intubation seems to reduce the risk of aspiration. As for traumatic patients, only one study explicitly considered the risk of aspiration. Davis et al. found no increased incidence in either patient group. Studies in traumatic patients have focused more on mortality, which appears to be primarily related to a reduced GCS, rather than intubation itself. For non-traumatic pediatric patients, intubation seems to be a factor related to a worse prognosis.

Internationally, it is commonly taught that a trauma patient who presents with a GCS score less than or equal to 8, should receive advanced and definitive management of the airways, i.e., orotracheal intubation [1]. The results of our systematic review challenge this dogmatic approach to airway management in patients with reduced consciousness. Although the GCS score could be associated with the risk of aspiration, especially in some retrospective studies [25, 26], the association between the presence or absence of protective airway reflexes and GCS score is not as firmly established [26, 27]. A study by Rotherhay et al. demonstrated that vomiting and cough reflexes decrease with a progressive decrease of GCS score in critically ill patients. However, the gag reflex appears to be a poor predictor of the need for intubation because it is absent in 20% of patients who have a GCS score of 15 [26]. Moulton et al. found that, on 111 patients with different levels of consciousness, as many as 14% of patients with a GCS score greater than 8 had an absent gag reflex (and 32% at least attenuated) [28].

Similarly, the evaluation and quantification of the reduction of consciousness in individual patients is not without controversy. The GCS is a tool invented in 1974 by Teasdale and Jennett to communicate long-term coma patients’ neurological course with a brain injury [29]. Although it is widely used to classify TBI’s severity, GCS was not initially designed for acute emergencies: numerous limitations have been highlighted over the years [30]. For example, to mention the largest, not all three scales that make up the GCS have the same predictive power [31, 32]. Besides, studies have shown that the GCS is not as highly reliable [33–35].

Furthermore, in the non-traumatic setting, different pathologies that determine the reduction of consciousness level can have different prognoses. Metabolic causes such as hypoglycemia lead to a dramatic decrease in the GCS score but are quickly resolvable by applying the prompt treatment. In the study by Nielsen et al., conducted on non-traumatic unconscious patients managed in the pre-hospital setting, 85% of patients regained consciousness before reaching the hospital [6].

Ultimately, the relationship between the level of consciousness (and the GCS) and intubation need is not yet sufficiently subject to evidence-based medicine. The studies that have tried to answer this clinical question are few and often of non-optimal quality: no randomized clinical trial has been conducted to date to answer the issue. The studies we have identified deal with two types of patients: intoxicated (or poisoned) patients and TBI patients. For the first ones, at least for the few prospective studies in the literature, not to proceed with intubation does not seem to increase the risk of aspiration [8, 16]. Nevertheless, some retrospective studies came to opposite conclusions: the risk of aspiration appears to be increased in non-intubated patients [17, 18].

While for traumatic patients, low GCS scores seem to be associated with a worse outcome, the intubated on-scene patients seem to have a higher mortality risk. This result appears to be contradictory: the severity of the brain injury can affect patients’ prognosis [36]. Thus, TBI patients with the lowest GCS score have a worse outcome, and the hypothesis that intubation of these patients will be detrimental or associated with multiple complications may not be easy to test. In this context, trials are needed that can exactly establish the role of intubation.

In summary, there is considerable uncertainty in the current literature regarding the impact of prevention of aspiration and mortality benefits from intubation for patients with reduced consciousness [37, 38]. Based on our review, it seems likely that the coma state’s etiology, rather than the GCS score alone, determines the aspiration risk and the consequent risk of pneumonia or pneumonitis. Whether intubation results in a reduction in the risk of comatose patients’ aspiration remains to be determined. It is also not yet determined whether these events and their complications lead to increased mortality.

### Limitations

As we have previously pointed out, there are no randomized controlled clinical trials in the literature that have addressed the clinical question examined by us. Moreover, the study design varies from study to study and does not always seem adequate to answer the clinical question. Even the outcomes considered are different and not adequate to exclude confounding bias, especially in retrospective studies. For the extreme heterogeneity of the studies (especially for the considered population and outcomes) and their low reliability, we considered a quantitative synthesis of the results via a meta-analysis not feasible.

Furthermore, we are aware that reducing consciousness is not the only parameter for intubating a traumatic or non-traumatic patient. However, we identified studies that respond to our clinical question through our research, excluding studies that have dealt with intubation as a necessary procedure in respiratory failure, not responding to non-invasive ventilation techniques.

## Conclusion

Although some prospective studies in non-traumatic comatose patients indicate that non-intubated patients are not at increased risk of aspiration than intubated patients, other retrospective studies have yielded opposite results. The few studies present for traumatized patients indicate that non-intubated patients do not present an increased risk of aspiration, but even in this case, the results of the different studies are conflicting.

For the currently available evidence, whether the intubation determines a reduction in aspiration events incidence and if these are more frequent in patients with low GCS scores are not yet established. The paucity of evidence on this topic makes clinical trials justifiable and necessary.

## Supplementary Information

**Additional file 1.**

## Data Availability

Data are available following a reasoned request.
