# Supplementary material for: Endotracheal intubation to reduce aspiration events in acutely comatose patients: a systematic review
Source: Scand J Trauma Resusc Emerg Med. 2020 Dec 10;28:116. doi: 10.1186/s13049-020-00814-w (PMC7726605; doi:10.1186/s13049-020-00814-w)
Supplement: Supplementary file 1 — Additional file 1. [file 13049_2020_814_MOESM1_ESM.docx]

**Pubmed**

*Pubmed searched on 8/10/2020 (5,187 results)*

(“Intubation” [mh] AND “Aspiration” [mh] AND “Glasgow Coma Scale” [mh] AND “Unconscious” [mh] OR “Comatose” [mh] AND “Intubation” [tiab] AND “Aspiration” [tiab] AND “Glasgow Coma Scale” [tiab] AND “Unconscious” [tiab] OR “Comatose” [tiab])

**Embase**

*Embase searched on 12/10/2020* ***(****10,255 results)*

(“Intubation” AND “Aspiration” AND “Glasgow Coma Scale” AND “Unconscious” OR “Comatose” AND “Incidence” AND “Acute”)

Sort by “Relevance”

**Cochrane Central Register of Controlled Trials (CENTRAL)**

*CENTRAL searched via Cochrane Library Interface on 13/10/2020 (17 citations)*

1 MeSH descriptor “intubation” AND “aspiration”, explode all trees

2 (“intubation”): ti,ab,kw AND (“aspiration” ):ti,ab,kw AND (“unconscious”):ti,,ab,kw

**Scopus Library (Elsevier)**

*Searched on 11/10/2020 (29,575 results)*

1 MeSH descriptor “Intubation” and “Aspiration”, explode all trees.

**SpringerLink**

*Searched on 14/10/2020 (9,561 results)*

1 MeSH descriptor “Intubation” and “Aspiration”, explode all trees.

**Ovid Emcare**

Searched on 14/10/2020 (0 results)

1 MeSH descriptor “Intubation” and “Aspiration”, explode all trees.
